# miR-126-3p containing exosomes derived from human umbilical cord mesenchymal stem cells promote angiogenesis and attenuate ovarian granulosa cell apoptosis in a preclinical rat model of premature ovarian failure

**DOI:** 10.1186/s13287-022-03056-y

**Published:** 2022-07-26

**Authors:** Qingxi Qu, Linghong Liu, Yuqian Cui, Hongli Liu, Jingyang Yi, Weidong Bing, Chunxiao Liu, Detian Jiang, Yanwen Bi

**Affiliations:** 1 Department of Obstetrics and Gynecology, Qilu Hospital of Shandong University, Jinan, 250012 People’s Republic of China; 2 Research Center of Stem Cell and Regenerative Medicine, Shandong University, Jinan, 250012 People’s Republic of China; 3 Laboratory of Cryomedicine, Qilu Hospital of Shandong University, Jinan, 250012 People’s Republic of China; 4 Otago Medical School, Christchurch Hospital, University of Otago, Christchurch, 8011 New Zealand; 5 Department of Cardiovascular Surgery, Qilu Hospital of Shandong University, Jinan, 250012 People’s Republic of China

**Keywords:** Exosomes, Mesenchymal stem cells, Granulosa cell, Angiogenesis, Apoptosis, Premature ovarian insufficiency

## Abstract

**Background:**

In our previous research, we found that overexpression of miR-126-3p in human umbilical cord MSCs (hucMSCs) promoted human umbilical vein endothelial cells angiogenic activities through exosome-mediated mechanisms. The present study aimed to investigate the role of miR-126-3p-modified hucMSCs derived exosomes (miR-126-3p-hucMSCs-exosomes) on the treatment of premature ovarian failure (POF).

**Methods:**

Primary hucMSCs were isolated from human umbilical cords and identified by differentiation experiments and flow cytometry. miR-126-3p-hucMSCs were obtained by miR-126-3p lentivirus infection. miR-126-3p-hucMSCs-exosomes were purified by ultracentrifugation method and characterized by transmission electron microscopy and western blot analysis. Primary rat ovarian granulosa cells (OGCs) were collected from ovarian tissues and identified by cell immunohistochemistry. The effects of miR-126-3p-hucMSCs-exosomes and miR-126-3p on OGCs function were determined by cell proliferation and apoptosis assays in a cisplatin induced POF cell model. The levels of suitable target genes were analyzed by PCR and Western blot analysis and subsequent Dual-Luciferase reporter assay. The signal pathway was also analyzed by western blot analysis. A cisplatin-induced POF rat model was used to validate the therapeutic effects of miR-126-3p-hucMSCs-exosomes to treat POF. Ovarian function was evaluated by physical, enzyme-linked immunosorbent assay, and histological examinations in chemotherapy-treated rats. The angiogenesis and apoptosis of ovarian tissues were assessed by immunohistochemical staining and Western blots.

**Results:**

Primary hucMSCs and miR-126-3p-hucMSCs-exosomes and primary rat OGCs were successfully isolated and identified. The cellular uptake experiments indicated that miR-126-3p-hucMSC-exosomes can be internalized into OGCs in vitro. Annexin V-FITC/PI staining and EDU assays revealed that both miR-126-3p-hucMSCs-exosomes and miR-126-3p promoted proliferation and inhibited apoptosis of OGCs damaged by cisplatin. PCR and western blot analysis and subsequent dual-luciferase reporter assay verified that miR-126-3p targets the sequence in the 3' untranslated region of PIK3R2 in OGCs. Further analysis showed that PI3K/AKT/mTOR signaling pathway took part in miR-126-3p/PIK3R2 mediated proliferation and apoptosis in OGCs. In rat POF model, administration of miR-126-3p-hucMSCs-exosomes increased E2 and AMH levels, increased body and reproductive organ weights and follicle counts, and reduced FSH levels. But more importantly, immunohistochemistry results indicated miR-126-3p-hucMSCs-exosomes significantly promoted ovarian angiogenesis and inhabited apoptosis in POF rats. Additionally, the analysis of angiogenic-related factors and apoptosis-related factors showed miR-126-3p-hucMSCs-exosomes had pro-angiogenesis and anti-apoptosis effect in rat ovaries.

**Conclusions:**

Our findings revealed that hucMSCs-derived exosomes carrying miR-126-3p promote angiogenesis and attenuate OGCs apoptosis in POF, which highlighted the potential of exosomes containing miR-126-3p as an effective therapeutic strategy for POF treatment.

## Introduction

Premature ovarian failure (POF), also known as primary ovarian insufficiency (POI), is a gynecological endocrine disease characterized by the cessation of ovarian function in women younger than 40 years old, which manifests as elevated gonadotropins and decreased estrogen levels. The etiology of the POF was mainly associated with genetics, immunologic factors, physical factors, chemical factors, biological factors and other idiopathic factors [1]. Most POF patients suffer from menstrual disorders, infertility, and perimenopause syndrome. Although there is limited understanding of the pathogenesis of POF, existing literature has suggested that the injury and apoptosis of ovarian granulosa cells (OGCs) plays a key role in the development of POF. Currently, there is no satisfying protective strategy to treat POF, it is of great importance to develop a novel therapeutic strategy for chemotherapy-induced POF treatment.

In the past ten years, mounting evidences have indicated the beneficial application of mesenchymal stem cells (MSCs) on the treatment of POF. Studies have noted that the transplanted MSCs exert their therapeutic effects mainly through the paracrine mechanism instead of direct cell replacement [2], and the MSCs can be used to treat POF mainly owing to its effects on OGC apoptosis [3]. However, there are certain limitations to the clinical application of MSCs, such as potential immunogenicity or tumorigenicity [4].

Exosome, a nano-sized vesicle (10–100 nm in diameter) released from a variety of cells, severed as a critical messenger for intracellular communication by transferring bioactive lipids, nucleic acids, and proteins [5]. Exosomes have been identified as an important component in MSC paracrine secretion, more and more studies have revealed that MSC-derived exosomes (MSC-exosomes) can be used as an alternative therapy for the paracrine actions of MSCs including the treatment of POF [4] MSC-derived exosomes hold great potential for cell-free therapies because they have a similar biological function to their mother cells and have no obvious adverse effects of direct cell usage [6]. A previous study in our laboratory had shown that exosomes derived from human umbilical cord mesenchymal stem cell (hucMSCs-exosomes) exerted anti-apoptotic effects in OGCs in vitro [7].

MicroRNAs (miRNAs) is an extremely important part of the exosomal cargo. The functions of miRNAs are carried out through degradation or translational inhibition of the target mRNA at the posttranscriptional level, which is mediated by the complementary combination of the 3′ untranslated region of target mRNAs [8]. Our previous study has confirmed miR-126-3p as a positive regulator in the angiogenic activities, and its overexpression can significantly enhance the endothelial cell proliferation by targeting SPRED-1and PIK3R2, suggesting that miR-126-3p‐mediated therapy might be a potential therapeutic target for the paracrine actions of MSCs [9]. However, the role of miR-126-3p in OGCs is not very clear.

Recently, we demonstrated that hucMSC-exosomes can be up-taken by endothelial cell and promote its angiogenic activities in vitro [10]. Moreover, we obtained data that miR-126-3p can be packed into hucMSCs-exosomes and overexpression of miR-126-3p in hucMSCs can further enhance its vascular protective effects by exosomes mechanism [11]. However, the effect of miR-126-3p-overexpressed hucMSCs-exosomes in ovarian function and structure remains unclear. Based on our previous reports and findings, we aimed to explore whether miR-126-3p-modified hucMSCs derived exosomes (miR-126-3p-hucMSCs-exosomes) have protective effects on both ovary angiogenesis and OGCs function in POF model in this study.

## Materials and Methods

### Isolation and Culture of hucMSCs

The experimental procedures strictly followed the guidelines of the Ethics Committee of Qilu Hospital of Shandong University. Human umbilical cords were obtained from the informed, consenting delivery women, and the isolation and culture of hucMSCs were conducted based on the standard procedure as we have previously described [10, 11]. The hucMSCs were maintained in α-MEM (SH30265.01B, Hyclone, USA) containing 10% FBS (16140-071, Gibco, USA) and passaged by trypsin (25200-056, Gibco, USA). To confirm the identity of the isolated cells as MSCs, the expression of typical markers of MSCs, such as CD29, CD44, CD31, CD45, CD166, CD90 and CD105, was examined by flow cytometry. The multidirectional differentiation potential of hucMSCs into osteocytes and adipocytes was evaluated by using a commercially available differentiation kit (CHEM2000, ChemBio, China) according to the manufacturer’s recommendations. To construct the cell lines for stable and high expression of miR-126-3p, we selected lentivirus as overexpression vector in this study. The hucMSCs were transfected with miR-126-3p and control sequences by lentiviral vectors (Biolink, China) as we have reported [11]. The transfection efficiency of vectors was verified by GFP fluorescence signal and PCR.

### Isolation and Culture of GCs

Rat OGCs was isolated and cultured as described in a previous study [12]. In brief, female Wistar rats aged 3–4 weeks were anesthetized and sacrificed to harvest their ovaries, then preovulatory follicles in the ovaries were punctured for cell collection under a stereomicroscope. The obtained cells were washed and cultured in DMEM/F12 medium (Hyclone, USA) containing 10% fetal bovine serum (Gibco, USA) in a humidified atmosphere at 37 °C with 5% CO_2_. Hematoxylin–eosin (HE) staining and follicle-stimulating hormone receptor (FSHR) immunohistochemistry were employed for the characterization of OGCs. The following experiments were performed with OGCs at the first to third passages.

### Preparation of Exosomes

Exosomes were isolated and purified from hucMSCs supernatant by ultracentrifugation method as we have described [10]. We got 3 kinds of exosomes, named as hucMSCs-exosomes, GFP-hucMSCs-exosomes and miR-126-3p-hucMSCs-exosomes. The exosome pellets were resuspended in phosphate buffer saline (PBS) and protein content was determined by using BCA assay (P0010S, Beyotime, China). The morphology of hucMSCs-exosomes was captured by a transmission electron microscopy (TEM, Hitachi H-800, Japan) and identified by an experienced engineer of Shandong Normal University. Western blotting was also used to identify exosomes markers, such as TSG101, Hsp70, CD63, and calnexin. For exosomes uptake experiment, the extracted exosomes were labeled using PKH26 (red) according to manufacturer’s instructions and co-cultured with OGCs at a concentration of 100 μg/ml for 48 h. Cellular uptake of exosomes was observed by a fluorescence microscope (Zeiss, Germany).

### Flow Cytometry and EDU Assay

Cell apoptosis were evaluated by flow cytometry analysis utilizing Annexin V-FITC/PI apoptosis detection kit (BB-4101, Bestbio, China). In short, OGCs were harvested and washed, and then stained with Annexin V-FITC and PI in the darkroom, followed by flow cytometry, and analyzed using Flow Jo 7.6 software.

Cell proliferation was assessed by EDU incorporation assay with an EDU imaging kit (C10310-1, RiboBio, China), as described in our previous study [9]. In short, EDU reagent was added to OGCs in each well for 2 h incubation, followed by fixation, permeabilization, EDU staining, and DAPI counterstaining. The results of proliferation rate were analyzed by the percentage of EDU-stained OGCs to DAPI-stained OGCs.

### Dual Luciferase Reporter Assay

To verify whether miR-126-3p was targeted at PIK3R2 in OGCs, luciferase reporter assay was performed as we have previously described [13]. PIK3R2 sequence including wild type (WT) or mutant type (MUT) of miR-126-3p binding sites were cloned downstream of the firefly luciferase gene in pGL3 Luciferase reporter vectors. The PIK3R2 wild type vector (PIK3R2-WT) and mutant type vector (PIK3R2-MUT) were co-transfected with miR-126-3p mimic or mimic NC into OGCs respectively. The cells were harvested after a 48 h period of transfection, and luciferase activity was measured using the Dual-Glo Luciferase Assay System.

### Establishment of POF Rat Model

All procedures of animal experiments were performed according to the Guide for the Care and Use of Laboratory Animals. Five-week-old Wistar female rats were obtained from Experimental Animal Centre of Shandong University. All the animals were weighed and measured serum levels of FSH, E2, and AMH before the cisplatin treatment. The POF rat model was established by daily intraperitoneal injections of cisplatin (1 mg/kg, dissolved in saline) for 14 days, as referred to a literature with a bit modification [14]. A single dose of 400 μg exosomal proteins suspended in 200 μl PBS was injected via caudal vein after 14 days of injection of cisplatin.

Five groups were divided randomly, *n* = 21 for each group. The first group was the normal group and received no treatment. The second group, the POF group, received two weeks intraperitoneal injection of cisplatin. The third group, the hucMSCs-exosomes group, received intraperitoneal injection of cisplatin and tail vein injection hucMSCs-exosomes. The fourth group, the miR-126-3p negative control hucMSCs- exosomes group (NC-hucMSCs-exosomes group), received intraperitoneal injection of cisplatin and tail vein injection miR-126-3p negative control hucMSCs-exosomes (NC-hucMSCs-exosomes). The fifth group, the miR-126-3p-hucMSCs-exosomes group, received intraperitoneal injection of cisplatin (Qilu-pharma, China) and tail vein injection miR-126-3p-hucMSCs-exosomes. The body weight was recorded at 0th, 1st, 2nd, 3rd and 4th week after exosomes treatment. Four weeks following exosomes treatment, the rats were euthanized for further analysis.

### Enzyme-linked Immunosorbent Assay (ELISA)

All the animals were measured serum levels of anti-Mullerian hormone (AMH), estradiol (E2), and follicle stimulating hormone (FSH) before the cisplatin treatment and 0th, 1st, 2nd, 3rd and 4th week after exosomes treatment. Blood samples were collected from retroorbital vein, and serum was separated and stored at − 20 °C until used for ELISA analysis. Serum levels of FSH (E-EL-R0026c, Elabscience, China), E2 (E-EL-0152c, Elabscience, China), and AMH (E-EL-R3022, Elabscience, China) were detected by ELISA kit according to the instructions provided by the manufacturer.

### HE Staining and Immunohistochemical Staining

Ovaries were collected after the rats were sacrificed and fixed in 4% paraformaldehyde, followed by dehydration in an ethanol gradient. After paraffin embedding, the tissues were sectioned into 6-µm thickness slices. The slices were stained with hematoxylin and eosin to observe the ovarian morphology and follicle counts.

For immunohistochemistry staining, the obtained paraffin sections were dewaxed in distilled water and incubated with the primary antibodies against proliferating cell nuclear antigen (PCNA, 1:200, Proteintech, China) or CD31(1:100, Proteintech, China) overnight at 4 °C. And then, sections were processed with corresponding secondary antibodies followed by horseradish peroxidase. Eventually, the sections were counterstained with hematoxylin and visualized with 3,3′-diaminobenzidine.

#### Apoptosis Assay

Ovarian tissue apoptosis was detected by the TUNEL apoptosis assay kit (E-CK-A322, Elabscience, China) according to the manufacturer’s instructions. Based on the instructions, the paraffin sections were deparaffinized, rehydrated and washed. Then, the slices were treated with protease K and stained with TUNEL reaction mixture. The nucleus was labeled by incubating sections with DAPI and the apoptosis staining signals were observed with a fluorescence microscope.

### RNA Isolation and Quantification

Total RNA was extracted from cells and ovarian tissues by using TRIzol reagent (15596–018, Thermo Fisher, USA) under the instruction of manufacturer. The concentration and quality of total RNA was analyzed by a Nano-Drop spectrophotometer. The RNA was reversely transcribed into cDNA according to the instructions of RNA reverse transcription kit and RT-PCR was carried out using the instrument of CFX96 Touch Real-Time PCR Detection System (BioRad, USA). U6 and GAPDH were used as an internal control, and the relative gene expression was calculated using the 2–ΔΔCt method.

### Western Blot Analysis

For western blot analysis, cells and ovarian tissues were collected and lysed with RIPA buffer. The protein concentration of each sample was quantified using a BCA Protein Assay Kit (P0010S, Beyotime, China). Equal amounts of proteins were separated on sodium dodecyl sulfate polyacrylamide gel electrophoresis (SDS-PAGE) and electroblotted onto fabrication of polyvinylidene fluoride (PVDF) membranes. The membranes were individually incubated with the specific antibodies, and then incubation with HRP-conjugated secondary antibodies. The enhanced chemiluminescence reagent was used to detect the target proteins. The antibodies used in this study included: PIK3R2 (1:10000, Abcam, USA), SPRED-1 (1:10000, Abcam, USA), calnexin (1:2000, Abcam, USA), AKT (1:2000, Cell Signaling, USA), p-AKT (1:2000, Cell Signaling, USA), ERK1/2 (1:2000, Cell Signaling, USA), p-ERK1/2 (1:2000, Cell Signaling), TSG101 (1:2000, Proteintech, China), HSP70 (1:2000, Proteintech, China), and CD63 (1:1000, Proteintech, China), and GAPDH (1:5000, Proteintech, China).

### Statistical Analysis

All of the experiments were repeated at least three times. All data were analyzed using GraphPad Prism version 8. Group differences were performed by Student’s *t* test or ANOVA for two groups or among multiple groups comparison. All quantitative data are shown as the mean ± standard deviation (SD). *P* value less than 0.05 was considered statistically significant.

## Results

### Identification of hucMSCs and hucMSCs-exosomes

**Fig. 1:**
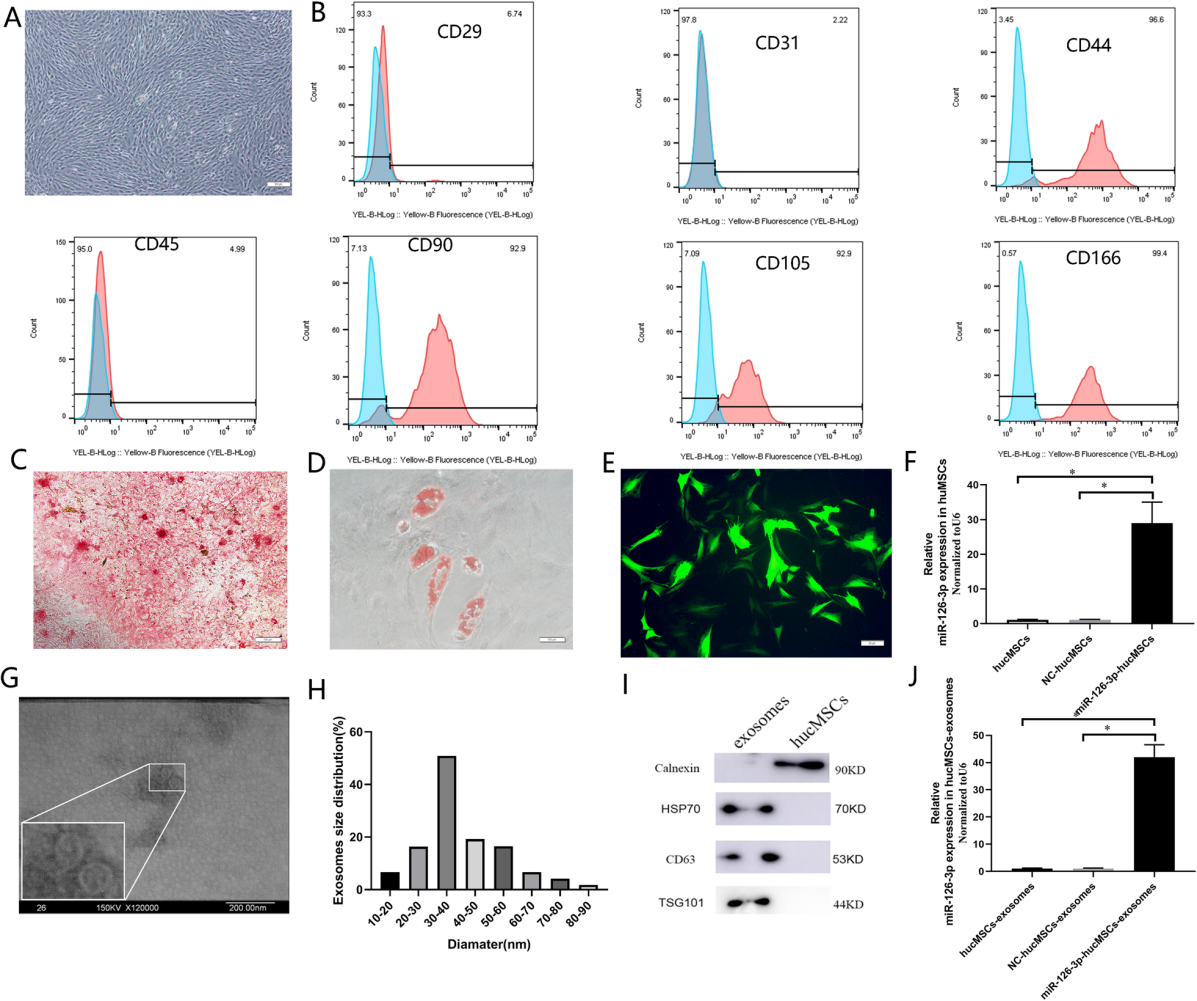
Identification of hucMSCs and hucMSCs-exosomes. **A**. Primary hucMSCs exhibited a typical fibroblastic morphology 14 days after culture (40). **B**. The cells were positive for CD29, CD44, CD166, CD90 and CD105 and negative for CD34 and CD45. **C**. hucMSCs differentiated into osteocytes by alizarin red staining (100). **D**. hucMSCs differentiated into adipo-lineage cells by oil red o staining (200). **E**. Expression of green fluorescent protein in miR-126-3p–transfected hucMSCs (200). **F**. The expression of miR-126-3p in hucMSCs-exosomes was detected by PCR **G**. Morphology of isolated exosomes using TEM. **H**. The mean diameters of the isolated exosomes. **I**. Expression levels of TSG101, HSP70, CD63, and calnexin in hucMSCs-exosomes were detected by western blot. **J**. The expression of miR-126-3p in hucMSCs-exosomes was detected by PCR. *P < 0.05 for all figures

It was observed that hucMSCs exhibited a typical fibroblast-like morphology (Fig. 1A). They strongly expressed CD29, CD44, CD90, CD105, and CD166, but the expression of CD31and CD45 were negative, as shown by Flow analysis (Fig. 1B). The ability of hucMSCs to induce differentiation was tested in vitro. They were positive for the osteocyte staining by Alizarin Red and adipocyte staining by Oil Red O (Fig. 1C and D). Lentiviral vectors carrying miR-126-3p was transfected into hucMSCs (Fig. 1E), resulting in a stable expression of exogenous miR-126-3p genes (29-fold vs. hucMSCs, *P* < 0.05, Fig. 1F). Gradient ultracentrifugation method was used to extract exosomes from the culture medium of hucMSCs. Morphology of hucMSCs-exosomes were observed by TEM (Fig. 1G), which confirmed particle diameters of 10–100 nm (Fig. 1H). Western blotting analysis indicated that the isolated particles expressed exosome surface markers, including TSG101, HSP70, and CD63 proteins. However, calnexin was not expressed (Fig. 1I), which should be negative control marker for exosome. On the basis of these evidences, the isolated particles were considered to be exosomes. We further measured the miR-126-3p expression in exosomes derived from hucMSCs and miR-126-3p-hucMSCs.The PCR data indicated that miR-126-3p-hucMSCs-exosomes had a 45-fold higher miR-126-3p expression level than that in miR-126-3p-hucMSCs (Fig. 1J).

### miR-126-3p hucMSCs-exosomes Could be Internalized into OGCs

**Fig. 2:**
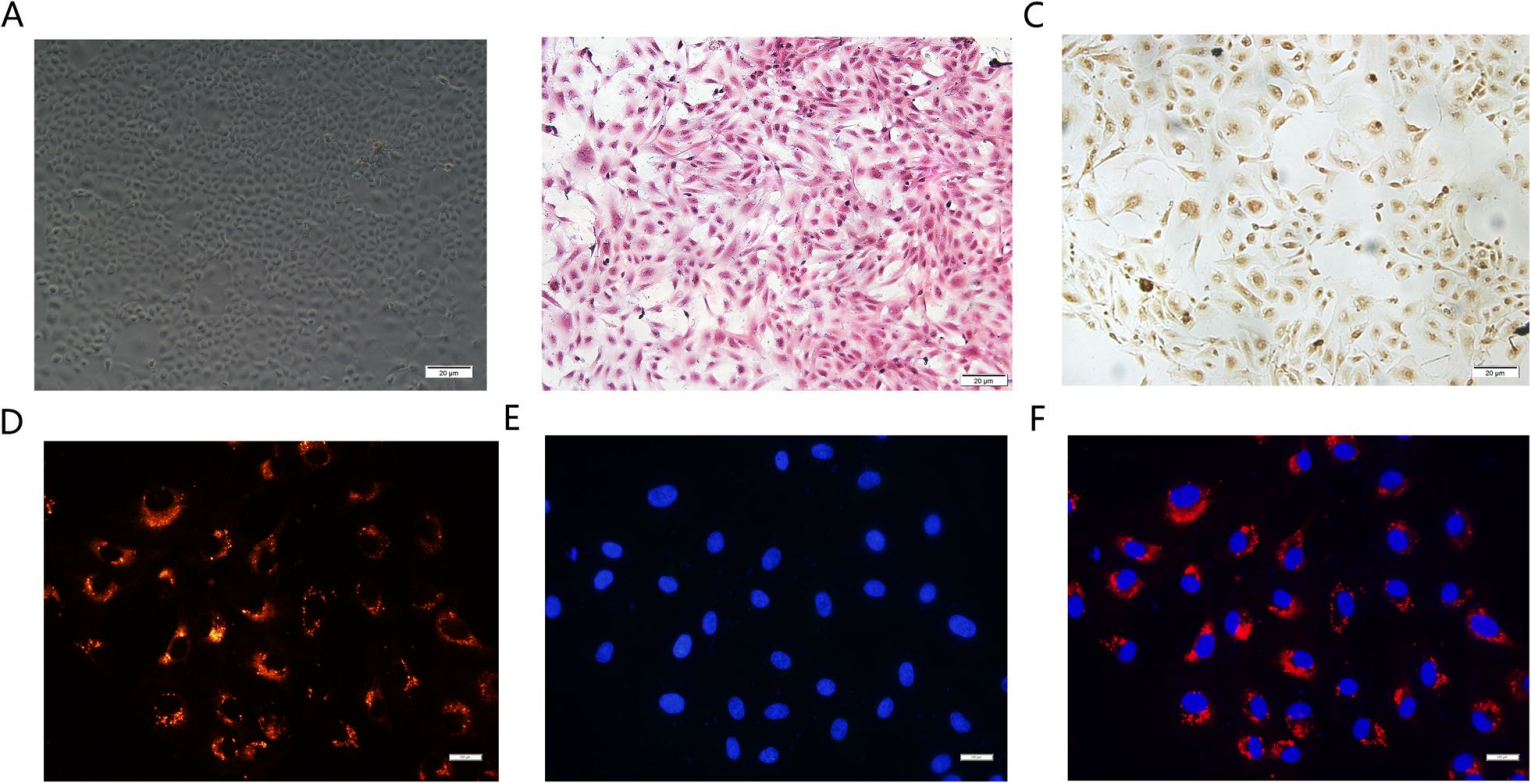
miR-126-3p hucMSCs-exosomes could be internalized into OGCs. **A**. The morphology of cultured OGCs **B**. Hematoxylin and eosin staining for identification of OGCs. **C**. Immunohistochemical staining of FSHR for identification of OGCs. **D**. The red stain indicated PKH26 -labeled miR-126-3p hucMSCs-exosome. **E**. The blue stain indicated the DAPI -labeled nucleus. **F**. The merged image of uptake of miR-126-3p hucMSCs-exosomes by OGCs

Primary rat OGCs were isolated and cultured from ovarian tissues (Fig. 2A). HE staining showed the typical granulosa shape of OGCs (Fig. 2B), immunohistochemical staining showed that the cultured mononuclear cells were positive for FSHR as reported previously (Fig. 2C) [15]. We then examined whether miR-126-3p-hucMSCs-exosomes could be internalized into OGCs. The miR-126-3p-hucMSCs-exosomes were labelled with the fluorescent reagent PKH26 and co-cultured with OGCs for 6 h. The red fluorescence in exosomes were observed in the perinuclear region of OGCs, implying that PKH26 -labeled exosomes can enter into the cytoplasm of OGCs (Fig. 2D–F). The phagocytosis phenomenon provided biological basis for the potential functional influence of miR-126-3p-hucMSCs-exosomes on OGCs.

### *miR-126-3p-hucMSCs-exosomes Improved the Proliferation Rate and Inhibited the Apoptosis Rate in POF OGCs *in vitro

**Fig. 3:**
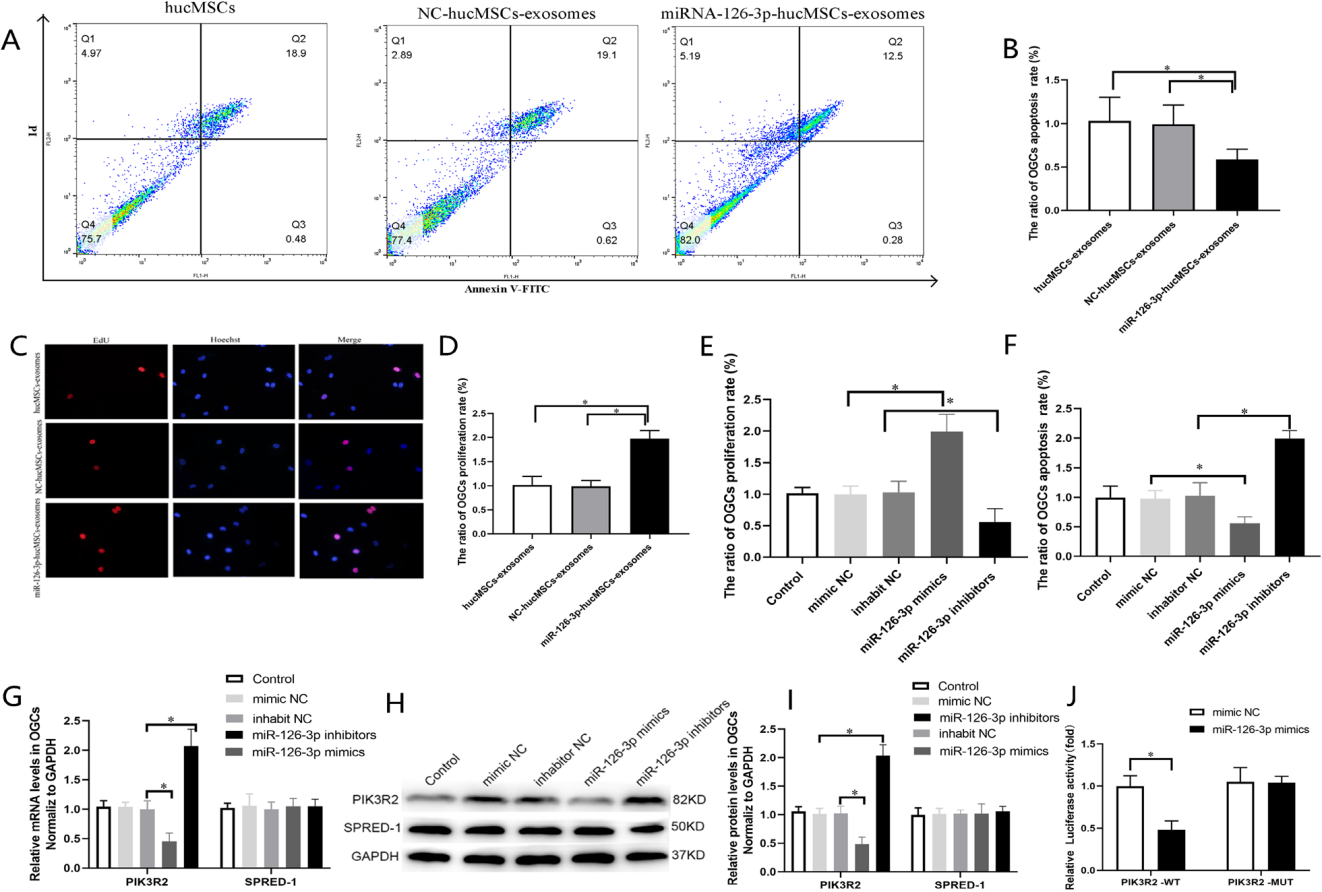
miR-126-3p promoted proliferation and inhibited apoptosis of OGCs in vitro and PIK3R2 is a target of miR-126-3p. **A**. Flow cytometric apoptosis assay was used to analyze the effect of miR-126-3p-hucMSCs-exosomes on OGCs apoptosis. **B**. Analysis of the ratio of the percentages of apoptotic OGCs in each group. **C**. EDU assay was used to analyze the effect of miR-126-3p-hucMSCs-exosomes on OGCs proliferation. **D**. Analysis of ratio of the percentages of proliferative OGCs in each group. **E**. Comparison of the ratio of apoptosis rate of OGCs as flow cytometric apoptosis assay in each group. **F**. Comparison of the ratio of proliferation rate of OGCs as EDU assay in each group. **G**. The mRNA levels of PIK3R2 and SPRED-1 after transfection of miR-126-3p mimics and inhibitor in OGCs. **H**. Representative western blots of PIK3R2 and SPRED-1 protein expression in OGCs transfected with miR-126-3p mimics and inhibitor. **I**. The protein levels of PIK3R2 and SPRED-1after transfection of miR-126-3p mimics and inhibitor in OGCs. **J**. After transfection of miR-126-3p mimic and wild-type or mutant vectors into OGCs, dual-luciferase reporter genes were performed to determine luciferase activity**P < 0.01 versus WT group. *P < 0.05 for all figures

To determine whether the changes of miR-126-3p in hucMSCs affected apoptosis of OGCs, we carried out a POF cell model using 4 µg/ml cisplatin to induce apoptosis of OGCs based on pre-tests of our laboratory [7].We conducted flow cytometry analyses using Annexin V-FITC/PI double-staining. As shown in Fig. 3A, the apoptosis rate of OGCs delivered with miR-126-3p-hucMSCs-exosomes remarkably decreased compared with hucMSCs-exosomes groups, suggesting that miR-126-3p-hucMSCs-exosomes suppressed OGCs apoptosis in vitro (Fig. 3B). Given the surprising positive results that we got in the apoptosis study, we further examined the effects of miR-126-3p-hucMSCs-exosomes on proliferation of OGCs. EDU assays showed that miR-126-3p-hucMSCs-exosomes significantly promoted GC proliferation (Fig. 3C and D).

### *miR-126-3p Improved the Proliferation Rate and Inhibited the Apoptosis Rate in POF OGCs *in vitro

Based on the above results, it is pretty well suspected that miR-126-3p played a positive role against cisplatin-induced injury of OGCs, at least in proliferation and apoptosis. To examine the functions of miR-126-3p for cell proliferation and apoptosis of OGCs, miR-126-3p mimics and inhibitors were transfected into OGCs cultured in vitro. EDU assays showed that miR-126-3p mimic significantly increased the proliferation rate of OGCs compared with mimic NC, but miR-126-3p inhibitor significantly decreased the proliferation rate of OGCs (Fig. 3E). Moreover, Annexin-V/PI staining assays revealed that miR-126-3p mimic significantly decreased the apoptosis rate of OGCs compared with mimic NC, but miR-126-3p inhibitor significantly increased the apoptosis rate of OGCs compared with inhibitor NC (Fig. 3F). Overall, these results indicated that miR-126-3p promoted proliferation and repressed apoptosis in POF OGCs in vitro.

### PIK3R2 is a Target of miR-126-3p

In our previous study, we have demonstrated that SPRED-1and PIK3R2 are targets of miR-126-3p in HUVECs and HSVECs [9, 11]. To elucidate the underlying molecular mechanisms responsible for the anti-apoptotic effects of miR-126-3p in OGCs, we firstly measured the expression of the SPRED-1and PIK3R2 levels by RT-PCR and western blot. Luckily, we found that transfection of miR-126-3p mimic markedly suppressed the mRNA and protein expression of PIK3R2 (Fig. 3G–I). By contrast, the mRNA and protein levels of PIK3R2 increased after miR-126-3p inhibitor transfection. However, we did not observe significant correlations between miR-126-3p and SPRED-1 expression after transfected miR-126-3p mimics or inhibitors into OGCs (Fig. 3G–I). The results suggested miR-126-3p may target PIK3R2 gene in OGCs. Therefore, we selected PIK3R2 for further study.

To further confirm whether miR-126-3p was targeted at PIK3R2 in OGCs, we performed the dual-luciferase reporter assay. As shown in Fig. 3J, compared with mimic NC in OGCs, the luciferase activity level of PIK3R2-WT was significantly reduced when responded to miR-126-3p mimics. However, the luciferase activity was not changed in PIK3R2-MUT by the miR-126-3p mimics. The results showed that miR-126-3p repressed the relative luciferase activities of the reporter gene by binding to the sequence in the 3' untranslated region of PIK3R2. These results therefore suggested that PIK3R2 were direct target genes of miR-126-3p in OGCs.

### Knockdown of PIK3R2 Promoted Proliferation and Inhibited Apoptosis of OGCs

**Fig. 4:**
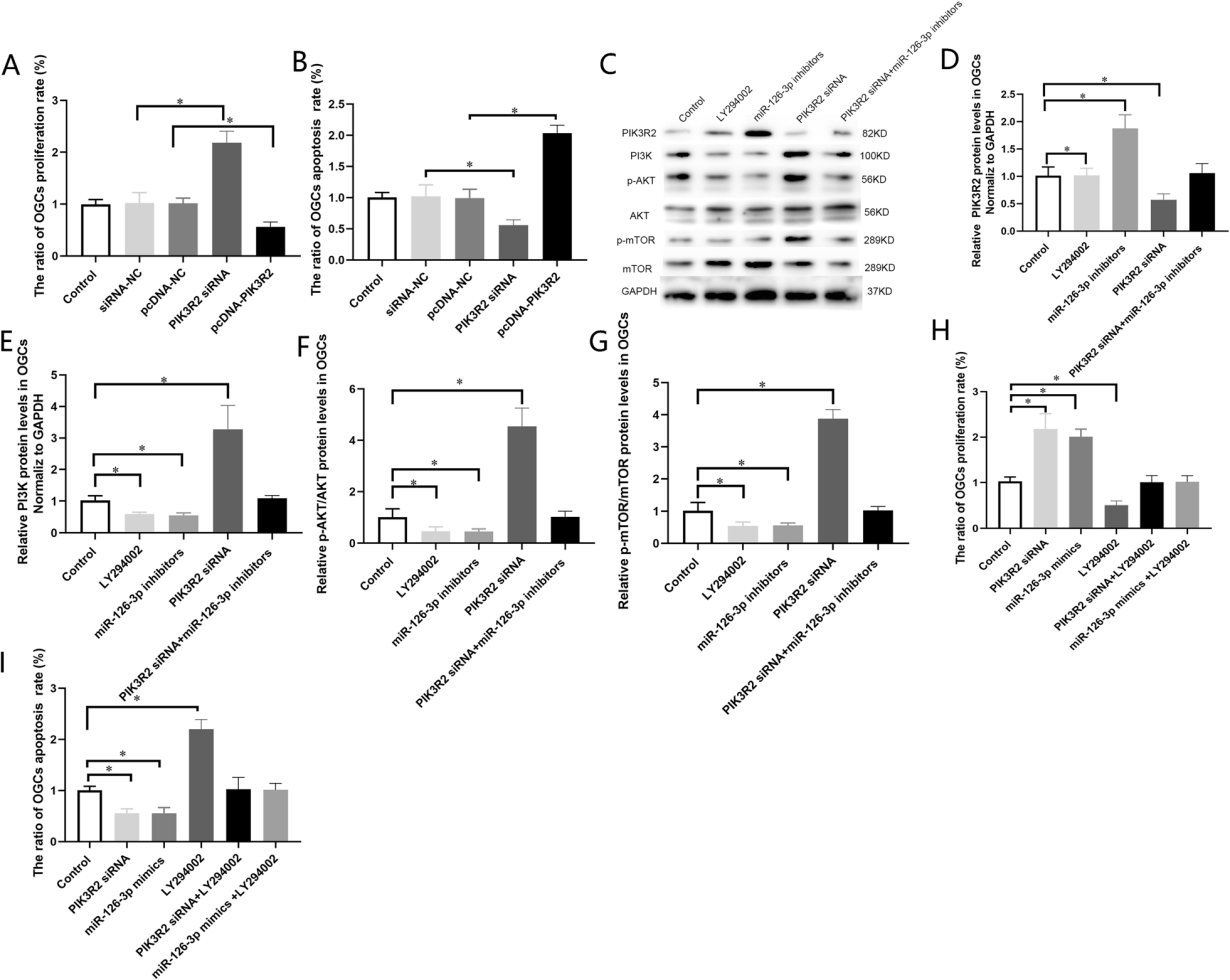
PI3K/AKT/mTOR signaling pathway involved miR-126-3p/PIK3R2 mediated OGC proliferation and apoptosis of OGCs in vitro **A**. Analysis of the ratio of the percentages of apoptotic OGCs in each group. **B**. Analysis of the ratio of the percentages of proliferative OGCs in each group. **C**. Representative western blots of PI3K/AKT/mTOR signaling pathway related proteins expression in OGCs in the different treatment group. **D**. The protein levels of PIK3R2 in OGCs in the different treatment group. **E**. The protein levels of PI3K in OGCs in the different treatment group. **F**. The protein levels of p-AKT in OGCs in the different treatment group. **G**. The protein levels of p-mTOR in OGCs in the different treatment group. **H**. Analysis of the ratio of the percentages of apoptotic OGCs in each group. **I**. Analysis of the ratio of the percentages of proliferative OGCs in each group. *P < 0.05 for all figures

**Fig. 5:**
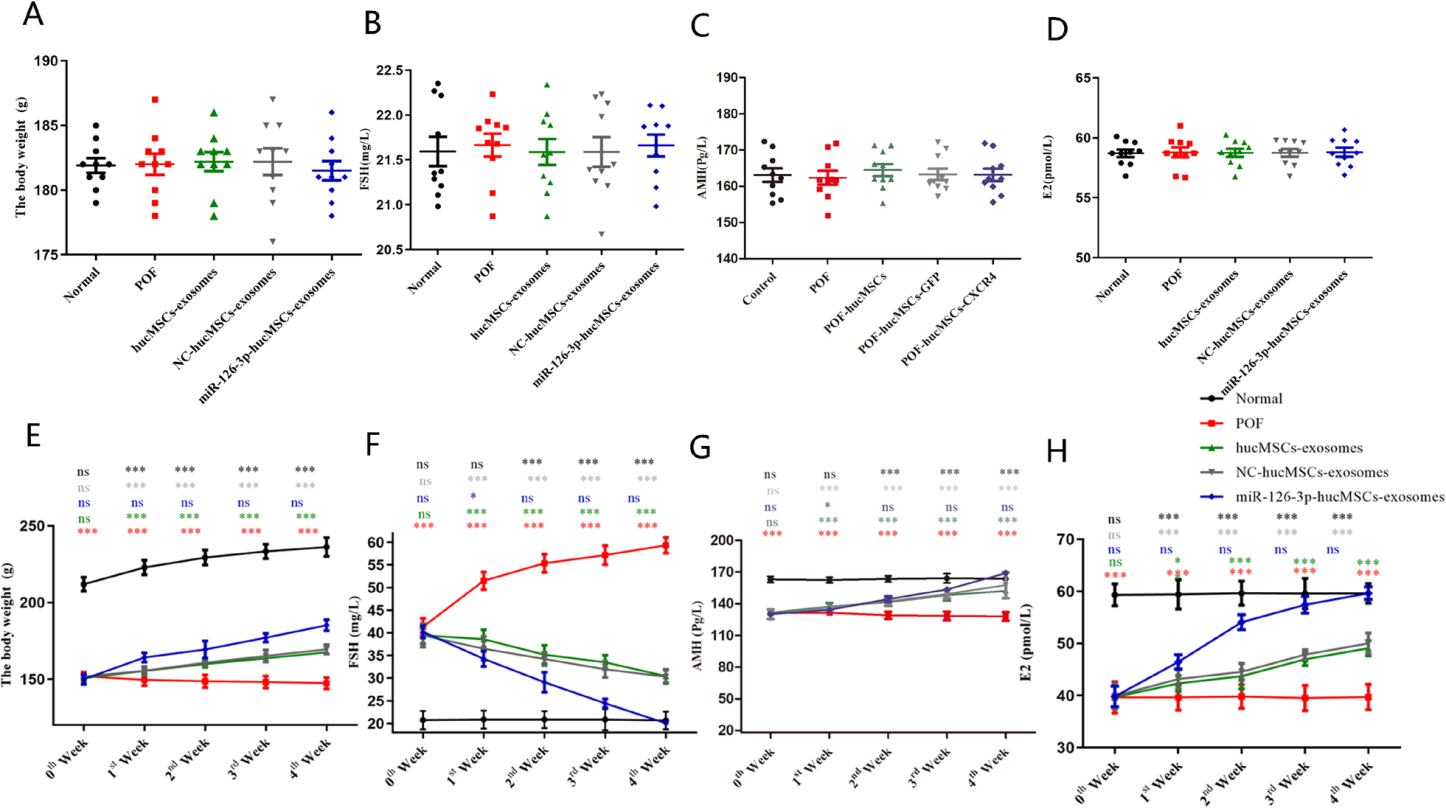
miR-126-3p-hucMSCs-exosomes treatment improved ovarian function in POF rat. The average values of body weight, FSH, AMH and E2 were record at the time before the cisplatin treatment and 0th, 1st, 2nd, 3rd and 4th week after exosomes treatment. **A**. Body weight in each group before the cisplatin treatment. **B**.FSH levels in each group before the cisplatin treatment. **C**. AMH levels in each group before the cisplatin treatment. **D**. E2 levels in each group before the cisplatin treatment. **E**. Body weight in each group during the experimental period. **F**. FSH levels in each group during the experimental period. **G**. AMH levels in each group during the experimental period. **H**. E2 levels in each group during the experimental period. Comparison of the body weight, FSH, AMH, and E2 levels between groups (POF vs. Normal, POF vs. hucMSCs-exosomes, hucMSCs-exosomes vs. NC-hucMSCs-exosomes, hucMSCs-exosomes vs. miR-126-3p-hucMSCs -exosomes, POF vs. miR-126-3p-hucMSCs-exosomes) at different times. The “ns” (*P* > 0.05) indicates no statistically significant difference. *P < 0.05, **P < 0.01, ***P < 0.001 for all figures. *n* = 10 for each group

To further confirm the functions of PIK3R2 expression for the cell proliferation and apoptosis of OGCs, OGCs were transfected with PIK3R2 siRNA and pcDNA3.1-PIK3R2. As shown in Fig. 5, the cell viability assay showed that knockdown of PIK3R2 promoted proliferation of OGCs (Fig. 4A), the flow cytometric apoptosis assay showed that knockdown of PIK3R2 inhibited apoptosis of OGCs (Fig. 4B). These results were consistent with the results of miR-126-3p mimic and miR-126-3p-hucMSCs-exosomes as afore mentioned. Moreover, when the PIK3R2 was over-expressed by pcDNA3.1- PIK3R2, the opposite experimental results on cell proliferation and cell apoptosis ability were identified (Fig. 4A and B). Thus, all these data strongly suggested down-regulation of PIK3R2 could promote proliferation and inhibit apoptosis of OGCs, and further demonstrated that PIK3R2 is a downstream target of miR-126-3p.

### PI3K/AKT/mTOR Signaling Pathway Involved miR-126-3p/PIK3R2 Mediated OGC Proliferation and Apoptosis of OGCs

In this study, we investigated whether the anti-apoptotic effects of miR-126-3p were due to PIK3R2-mediated PI3K/AKT/mTOR activation. OGCs were transfected with PI3K pathway inhibitor LY294002 or miR-126-3p inhibitor or PIK3R2 siRNA, the expression of PIK3R2, PI3K, AKT, and mTOR in OGCs were measured using Western blotting. The results of Western blot showed that, PIK3R2 siRNA resulted in increased the levels of PI3K and p-AKT and p-mTOR in OGCs (*P* < 0.01, Fig. 4C), where miR-126-3p inhibitor and LY294002 resulted in decreased levels of these proteins respectively. However, when miR-126-3p inhibitor and PIK3R2 siRNA were co-transfected into OGCs, no notable difference was found in levels of PIK3R2, PI3K, p-AKT, p-mTOR in OGCs (Fig. 4D–J). These results indicated that downregulation of PIK3R2 activated PI3K/AKT/mTOR pathway.

The cell proliferation and apoptosis of OGCs were detected after the cells were transfected with PIK3R2 siRNA and miR-126-3p mimic and LY294002. Both of PIK3R2 siRNA and miR-126-3p mimic promoted OGC proliferation and inhibited apoptosis (*P* < 0.01, Fig. 4H, I), when LY294002 and PIK3R2 siRNA were co-transfected into OGCs, the proliferation and anti-apoptotic effects of PIK3R2 siRNA were blocked. The same results were found in OGCs co-transfected miR-126-3p and LY294002. All these experimental results denoted that miR-126-3p promoted OGC proliferation and inhibited apoptosis by targeting PIK3R2 through PI3K/AKT/mTOR signaling pathway.

### miR-126-3p-hucMSCs-exosomes Treatment Improved Ovarian Function and Structure in Rats with Chemotherapy-induced Ovarian Damage

All the animals were weighed and measured serum levels of FSH, E2, and AMH before the cisplatin treatment, and no significant difference was observed between them (Fig. 5A–D). The administration of exosomes was conducted after 15 days of injection of cisplatin. The body weight was recorded at 0th, 1st, 2nd, 3rd and 4th week after exosomes treatment, and the levels of FSH, LH and E2 were detected by ELISA in serum at same time in each POF group. Our results showed that, the body weight and the levels of E2 and AMH in POF group were lower than that in the comparison group, and the FSH levels showed an opposite tendency. Moreover, compared to the POF group, the body weight and the levels of E2 and AMH in three exosomes groups increased at each point in time after treatment up to 4 weeks. However, the body weight and the levels of E2 and AMH were higher in the miR-126-3p-hucMSCs-exosomes group than in the NC-hucMSCs-exosomes group and hucMSCs-exosomes group, and the FSH levels showed an opposite tendency toward the levels of E2 and AMH again. Moreover, there was no significant difference in the body weight and the levels of E2 and AMH and FSH between the NC-hucMSCs-exosomes and hucMSCs-exosomes groups (Fig. 5E–H). The results showed that miR-126-3p-hucMSCs-exosomes could reduce histological damage by restore ovarian function more effectively.

**Fig. 6:**
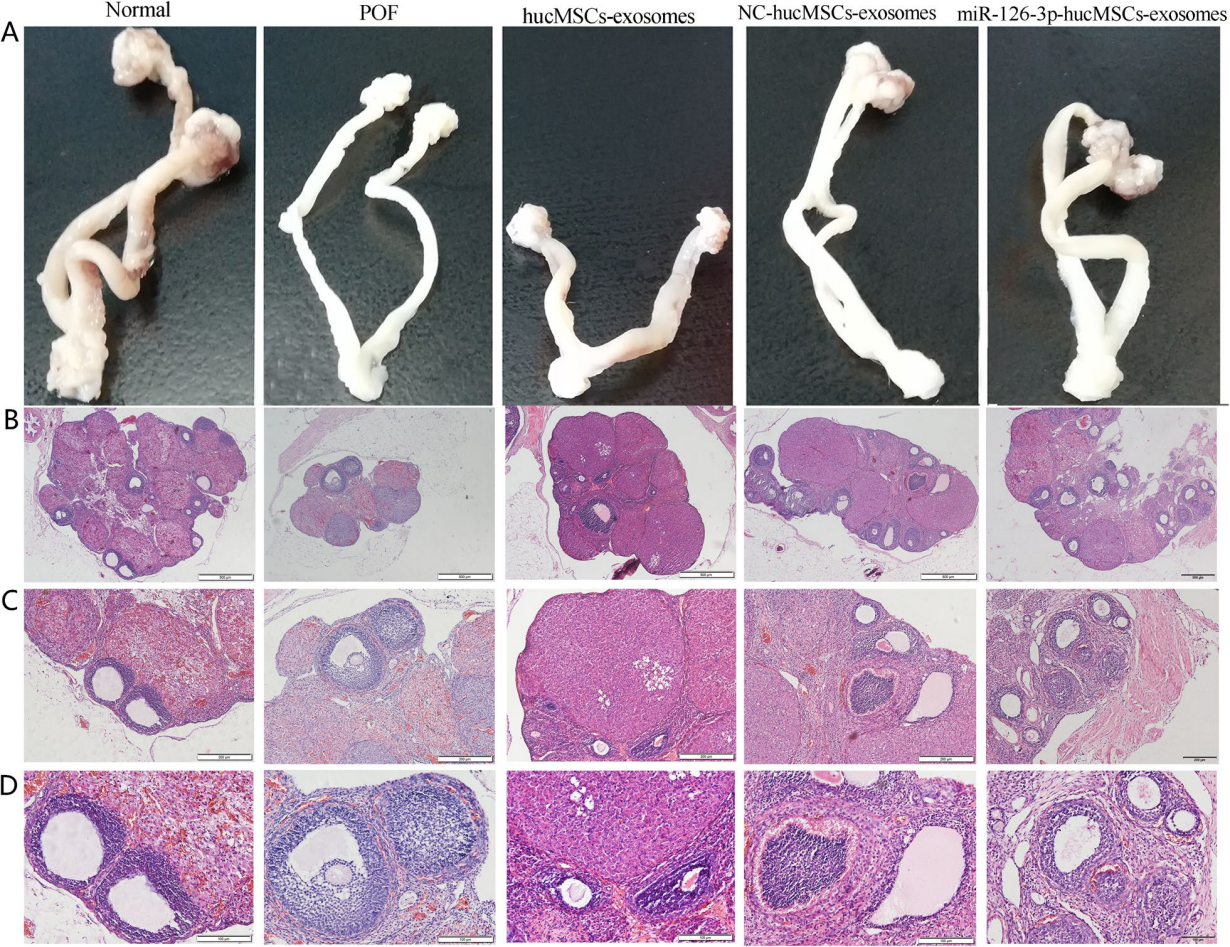
miR-126-3p-hucMSCs-exosomes treatment affected ovarian structure in rats with chemotherapy-induced POF. **A**. Representative photograph of rat reproductive organs in Normal group, POF group, hucMSCs-exosomes group, NC-hucMSCs-exosomes group, and miRNA-126-3p-hucMSCs-exosomes group. HE staining was performed to observe the histology changes and the number of follicles at different stages of rat ovaries at 4 weeks after miR-126-3p-hucMSCs-exosomes treatment (**B** × 40, **C** × 100, **D** × 200). *n* = 10 for each group

Ovaries and uteruses in each group were collected and subjected to pathological analysis at 4 weeks after the treatments. As presented in Fig. 6A, Ovaries and uteruses were highly modulated after the cisplatin treatment, and a marked increase in reproductive organ weight can be observed in exosomes treated groups, especially in miR-126-3p-hucMSCs-exosomes group. HE staining was performed to observe the histopathologic changes of the ovarian tissues. Results showed that many mature and healthy follicles were observed in the normal ovary tissues. In contrast, follicle count and corpora lutea count decreased and the morphologic structure was disordered in ovaries from the POF rats after cisplatin injection. As expected, the follicle count of the miR-126-3p-hucMSCs-exosomes group and NC-hucMSCs-exosomes group and hucMSCs-exosomes group increased compared to the POF group, and they were higher in the miR-126-3p-hucMSCs-exosomes group than in the NC-hucMSCs-exosomes group and hucMSCs-exosomes group. However, the counts of follicles at different developmental stages in the miR-126-3p-hucMSCs-exosomes group were still lower than those of the normal group (Fig. 6B–D). These results suggested that hucMSCs-exosomes overexpressing miR-126-3p could exert a stronger therapeutic effect in repairing damaged ovarian structures and improving function in rat models of POF.

### miR-126-3p-hucMSCs-exosomes Exerted Anti-apoptotic and Pro-angiogenic Effects in Rats with Chemotherapy-induced Ovarian Damage

**Fig. 7:**
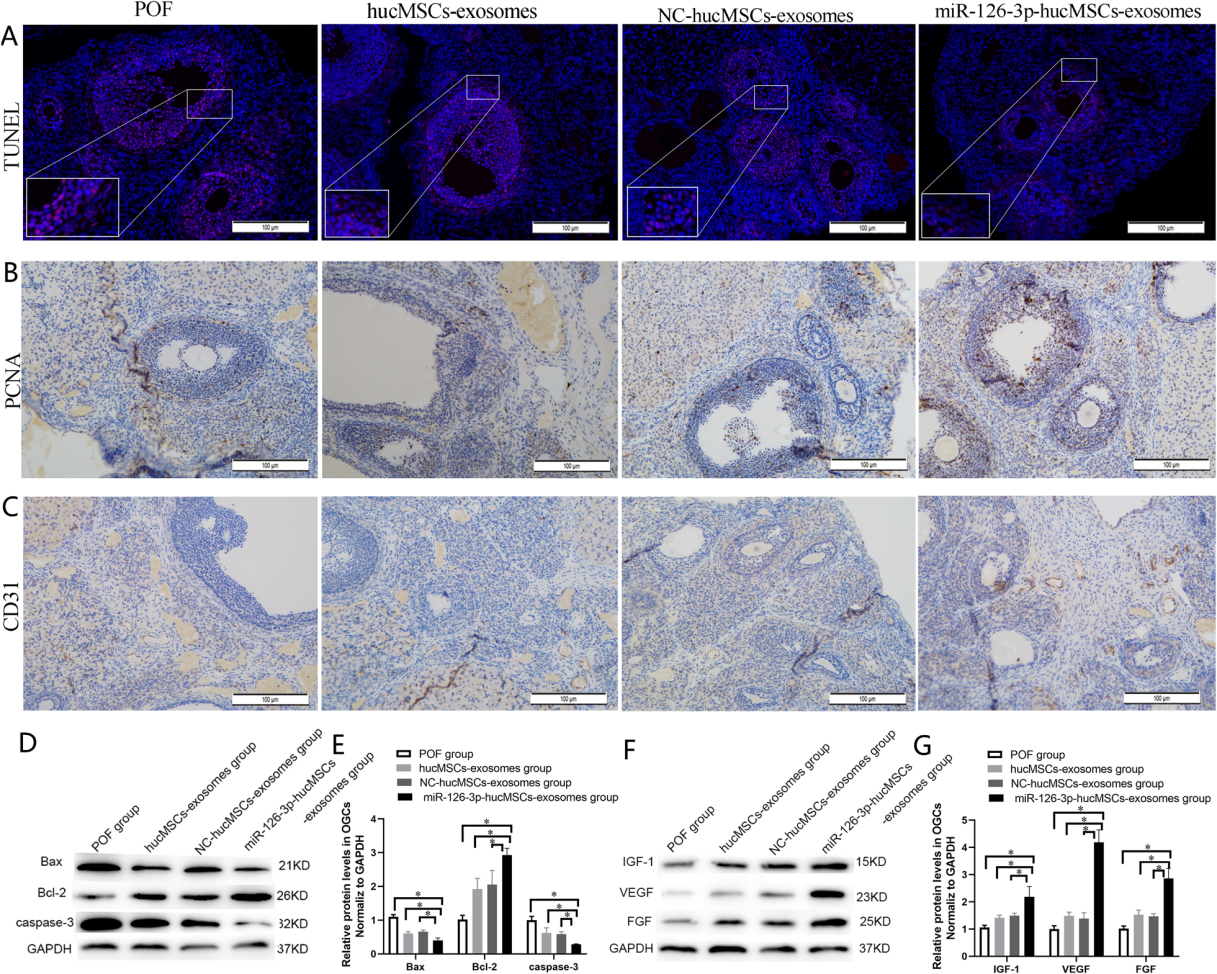
miR-126-3p-hucMSCs-exosomes exerted antiapoptotic and proangiogenic effects in rats with chemotherapy-induced POF. **A**. Representative images of the TUNEL apoptosis assay in the ovaries of each group (× 200). **B**. Representative images of PCNA immunohistochemistry staining in the ovaries of each group (× 200). **C**. Representative images of CD31 immunohistochemistry staining in the ovaries of each group (× 200). *n* = 10 for each group. **D**. Representative western blots of the angiogenic-related factors in the injured ovarian tissues at 4 weeks after miR-126-3p-hucMSCs-exosomes treatment. **E**. Quantification of the angiogenic-related factors in the different treatment group. **F**. Representative western blots of the apoptosis-related factors in the injured ovarian tissues at 4 weeks after miR-126-3p-hucMSCs-exosomes treatment. **G**. Quantification of the apoptosis-related factors in the different treatment group. *P < 0.05 for all figures. *n* = 10 for each group

Next, we checked whether the protective effects of miR-126-3p-hucMSCs-exosomes on ovarian function and structure were owing to its pro-angiogenic and anti-apoptotic effects. TUNEL staining was performed to evaluate the effects of hucMSCs-exosomes overexpressing miR-126-3p on OGCs apoptosis in the ovary tissues (Fig. 7A). Among the four cisplatin-injected groups, the apoptotic rate of the miR-126-3p-hucMSCs-exosomes group was lower than that of the POF group, hucMSCs-exosomes group and NC-hucMSCs-exosomes group. We also performed PCNA immunohistochemistry staining to detect OGCs proliferation in the ovary tissues (Fig. 7B). It was shown that the number of proliferating cells of the miR-126-3p-hucMSCs-exosomes group was higher than that of the POF group, hucMSCs-exosomes group and NC-hucMSCs-exosomes group. Ovarian angiogenesis in each group was assessed via CD31 immunohistochemistry staining (Fig. 7C). There was a significant increase in angiogenesis in miR-126-3p-hucMSCs-exosomes group in comparison with the hucMSCs-exosomes group and NC-hucMSCs-exosomes group. However, there was no significant difference between hucMSCs-exosomes and NC-hucMSCs-exosomes groups.

Western blot analysis was performed to detect the effect of exosomes on the release of apoptosis-related factors (Bax, Bcl-2, and caspase-3) and angiogenic-related factors (VEGF, IGF-1, and FGF) in the injured ovaries. As for apoptosis-related factors, miR-126-3p-hucMSCs-exosomes induced a significantly higher Bcl-2 and lower Bax and caspase-3 proteins expression than the other three POF groups (Fig. 7D, E). As for angiogenic-related factors, the representative expression levels were higher in the miR-126-3p-hucMSCs-exosomes group compared to the other three POF groups (Fig. 7F, G). All these results demonstrated that miR-126-3p-hucMSCs-exosomes exerted pro-angiogenic and anti-apoptotic effects in POF in vivo.

## Discussion

With the development of chemotherapeutic strategy, chemotherapeutic agents have been widely used to treat tumor patients in the past few decades, unavoidably cause the growing of the incidence of chemotherapy-induced POF cases. Although considerable progress has been made in treating POF, there is no radical cure to reverse the chemotherapy-induced ovarian damage. Although the molecular mechanism of ovarian dysfunction remains to be clarified, OGCs apoptosis was reported to be strongly associated with POF. In particular, vascular damage and ovarian cortex fibrosis are also involved in chemotherapy-induced POF. In recent years, stem cells-based therapies had attracted great attention as a way to restore ovarian function owing to its effects on OGCs apoptosis and follicular atresia [16]. Our study aimed to explore the role of miR-126-3p-hucMSCs-exosomes play on treatment of POF in vitro and in vivo.

**Fig. 8:**
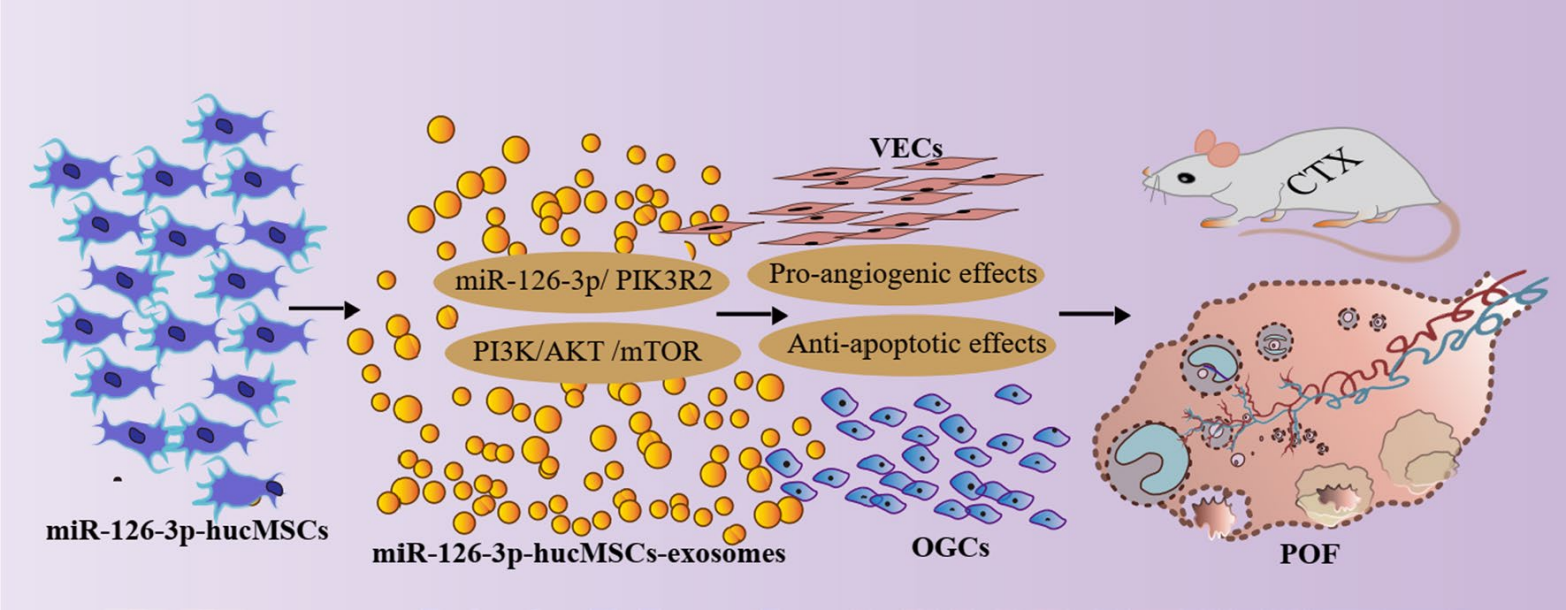
Graphical abstract of experimental procedures presented to study the effects and mechanisms of miR-126-3p-hucMSCs-exosomes on chemotherapy-induced POF

In this study, we firstly isolated hucMSCs and hucMSCs-exosomes and loaded miR-126-3p into hucMSCs-exosomes by genetic modification of hucMSCs. Then we isolated primary rat OGCs and investigated the role of miR-126-3p-hucMSCs-exosomes in OGCs biological behavior and possible mechanism. Next, we constructed a cisplatin-induced POF rat model and validate therapeutic effects of miR-126-3p-hucMSCs-exosomes on ovarian function and structure. Lastly, we showed the pro-angiogenic and anti-apoptotic effects of miR-126-3p-hucMSCs-exosomes in rats with chemotherapy-induced ovarian damage. The experimental procedures were presented in Fig. 8. Taken together, the present study is the first to demonstrate that hucMSCs derived exosomal miR-126-3p overexpressing can stimulate the recovery of damaged ovarian structures and function via promote angiogenesis and attenuate OGCs apoptosis in the setting of chemotherapy-induced POF.

In our previous research, we found that overexpression of miR-126-3p in hucMSCs promoted HUVECs proliferation, migration, and tube formation activities through exosome-mediated mechanisms in vitro. miR-126-3p-hucMSCs-exosomes can activate an angiogenesis program through targeting SPRED-1 and PIK3R2 and strengthen VEGF signal in vitro [9, 11]. Therefore, we hypothesized that miR-126-3p-hucMSCs-exosomes may have a therapeutic effect of POF through the pro-angiogenesis mechanism in vivo. Due to the major role of OGCs played in maintaining ovarian follicular development in the process of POF, we considered that it was also necessary to estimate the effects of miR-126-3p-hucMSCs-exosomes in damaged OGCs in vitro firstly. Therefore, we isolated and cultured primary OGCs from rat ovarian tissues and established a POF cell model in the present study. We observed that miR-126-3p-hucMSCs-exosomes can enter into OGCs and that improved the proliferation rate and inhibited the apoptosis rate in POF OGCs in vitro.

Angiogenesis plays a critical role in the ovarian development and recovery of ovarian function function [17]. Regulation of ovarian angiogenesis improves follicular development and restores ovarian function in POF [18]. miR-126-3p has been proved to be important to vascular integrity and angiogenesis [19, 20]. Our previous study had identified the role of miR-126-3p in vascular endothelial activities using gain- and loss-function studies [11].However, the role of miR-126-3p in OGCs function especially in cell apoptosis and proliferation has rarely been studied. On the other hand, a previous study in our laboratory had shown that hucMSCs-exosomes exerted an anti-apoptotic effect in OGCs in vitro [7]. To make clear the underlying molecular mechanisms by which miR-126-3p-hucMSCs-exosomes inhibited OGCs apoptosis and improved proliferation, we performed functional studies by transfected with miR-126-3p mimics or inhibitor in OGCs. Surprisingly, our in vitro results showed that miR-126-3p promoted proliferation and repressed apoptosis in cisplatin-induced injury of OGCs. In addition, we further demonstrated that miR-126-3p down-regulate PIK3R2 expression through post-transcriptional regulation by binding to the sequence in the 3' untranslated region of PIK3R2. Interestingly, in line with our study, another study by Xiaofeng Zhou showed that miR-126-3p were able to inhibit apoptosis by targeting PIK3R2 in porcine OGCs, which supports the results of this study [21].

It is well known that PIK3R2 is a regulatory factor of the PI3K/AKT/mTOR pathway [22, 23]. Meanwhile, the PI3K/AKT/mTOR pathway has been reported to be associated with cell apoptosis [24, 25]. In our study, miR-126-3p has been verified to specifically target PIK3R2 by dual luciferase reporter gene assay, which led us to hypothesize that miR-126-3p could promote OGC proliferation and inhibited apoptosis by targeting PIK3R2 through PI3K/AKT/mTOR signaling pathway. In the present study, we knocked down PIK3R2 in OGCs to determine the relationship between miR-126-3p and PI3K. As expected, our findings provided evidence that miR-126-3p could promote proliferation and inhibited apoptosis of OGCs via the PIK3R2-mediated PI3K/AKT/mTOR activation.

It had been reported that stem cell-based therapy could restore damaged ovarian function through improving the number of ovarian follicles through a paracrine mechanism [26, 27]. The present study and our previous study had shown the anti-apoptotic and pro-angiogenic effects of miR-126-3p in vitro [11]. Therefore, we hypothesized that hucMSCs-exosomes carrying miR-126-3p could produce synergistic effects for the prevention and treatment of POF in vivo. In our animal experiments, we first illustrated that the administration of miR-126-3p-hucMSCs -exosomes increased E2 and AMH levels, increased body and reproductive organ weights and follicle counts, and reduced FSH levels. We then performed immunostaining and TUNEL staining to measure angiogenesis and apoptosis of GCs in ovaries, all these results indicated miR-126-3p-hucMSCs-exosomes restored ovarian function by promote ovarian angiogenesis and inhabit apoptosis in POF rats. Besides, we also provided the evidence that administration of miR-126-3p-hucMSCs-exosomes elevated expression of the angiogenic-related factors (VEGF, IGF-1, and FGF) and diminished apoptosis-related factors (Bax and caspase-3) in rats with POF. All these findings are consistent with the results of the latest research showing that human amniotic epithelial cell-derived exosomes restore ovarian function by transferring microRNAs against apoptosis [28].

There are still some limitations in this study that could be improved. Firstly, the exosome extraction strategies in this study needs to be further developed in order to produce purer and higher volumes of membrane vesicles. Secondly, we only used morphologic and functional tests to evaluate ovary damage in this study. However, the evaluation of reproductive function in chemotherapy-induced POF rats is need to be addressed. Thirdly, we only determined PIK3R2 as a direct target gene of miR-126-3p in OGCs, later experiments will be examining whether there are any other downstream molecules associated with miR-126-3p. Finally, the research on the long-term effects of miR-126-3p-hucMSCs-exosomes remains insufficient, and the investigation is still in the preclinical stage. Therefore, more profound research is required for further evaluation of its efficacy and safety.

## Conclusions

In conclusion, we firstly demonstrated that the delivery of miR-126-3p via hucMSCs-derived exosomes acts to promote proliferation while inhibit the apoptosis of OGCs through PIK3R2/PI3K/AKT/mTOR axis in vitro. Combined with early experimental studies, we provided evidence that hucMSCs-derived exosomes carrying miR-126-3p improves ovarian function and structure integrity by promotes angiogenesis and attenuates OGCs apoptosis in a preclinical rat model of POF. The present study revealed that hucMSCs-exosomes carrying miR-126-3p could exert a synergistic effect and represented a promising future cell-free therapy for developing therapeutic treatments for POF.

## Data Availability

The data that support the findings of this study are available from the corresponding author upon reasonable request.

## Abbreviations

MSC: Mesenchymal stem cell
hucMSCs: Human umbilical cord MSCs
OGCs: Ovarian granulosa cells
hucMSCs-exosomes: Exosomes derived from human umbilical cord MSCs
TEM: Transmission electron microscopy
miR-126-3p-hucMSCs-exosomes: MiR-126-3p-modified hucMSCs derived exosomes
HUVEC: Human umbilical vein endothelial cell
HE: Haematoxylin and eosin
POF: Premature ovarian failure
POF: Primary ovarian insufficiency
E2: Estrogen
AMH: Anti-Mullerian hormone
FSH: Follicle stimulation hormone
ELISA: Enzyme-linked immunosorbent assay
PBS: Phosphate buffer saline
SDS-PAGE: Sodium dodecyl sulfate polyacrylamide gel electrophoresis
PVDF: Fabrication of polyvinylidene fluoride
PCNA: Proliferating cell nuclear antigen
